# The role of new carbapenem combinations in the treatment of multidrug-resistant Gram-negative infections

**DOI:** 10.1093/jac/dkab353

**Published:** 2021-11-21

**Authors:** Emilio Bouza

**Affiliations:** Department of Clinical Microbiology and Infectious Diseases, Hospital Gregorio Marañon, Department of Medicine Universidad Complutense, CIBERES, Ciber de Enfermedades Respiratorias, Madrid, Spain

## Abstract

Multi-drug resistant (MDR) Gram-negative bacteria represent a growing threat, with an increasing prevalence of carbapenem-resistant Enterobacterales (CRE) infections, for which treatment options are limited. New treatment combinations composed of a β-lactam antibiotic plus a potent β-lactamase inhibitor (BLI) with anti-carbapenemase activity have been developed, including two carbapenem/BLI combinations that are commercially available—meropenem/vaborbactam (Vabomere^®^ in the US, Vaborem^®^ in Europe; Melinta Therapeutics) and imipenem/cilastatin/relebactam (Recarbrio^®^; Merck Sharp & Dohme), plus one other (meropenem/nacubactam) in early clinical development. This review provides a summary of the preclinical evidence supporting the use of carbapenem/BLI combinations and presents the clinical evidence across a range of MDR Gram-negative infections, with a focus on the use of meropenem/vaborbactam. All three BLIs have shown *in vivo* activity against *Klebsiella pneumoniae* carbapenemase and other class A carbapenemases. In 2019, meropenem/vaborbactam was listed in the WHO’s list of essential medicines, because of its activity against priority 1 antibiotic-resistant pathogens. Meropenem/vaborbactam has considerable *in vitro and in vivo* activity against CRE, and *in vitro* evidence showing a low potential for resistance at clinically relevant doses. In randomized trials, meropenem/vaborbactam was non-inferior to piperacillin/tazobactam in patients with complicated urinary tract infection and more effective than the best-available treatment in patients with serious CRE infections. Meropenem/vaborbactam is well tolerated and, based on clinical experience, demonstrated lower toxicity compared with the combination regimens that have previously been the standard of care. In conclusion, carbapenem/BLI combinations represent an important therapeutic strategy in patients with MDR Gram-negative infections.

## Introduction

Major public health organizations around the world, including the WHO, US CDC, and the ECDC, recognize the growing threat of MDR Gram-negative bacteria.^1–3^ All of the priority 1 (critical) pathogens identified by the WHO in their 2017 report on antibiotic research priorities are Gram-negative MDR organisms (*Acinetobacter baumannii, Pseudomonas aeruginosa*, and Enterobacterales), and all but two of the nine priority 2 and 3 pathogens are Gram-negative MDR.^3^

Carbapenem antibiotics are the mainstay of treatment of MDR Gram-negative infections; they are recommended for serious infections caused by extended-spectrum β-lactamase (ESBL)-producing Enterobacterales, and are the drugs of choice for the empirical treatment of sepsis caused by MDR Gram-negative organisms.^4^ However, there is growing concern about an increase in carbapenem resistance among MDR Gram-negative organisms,^4^ and evidence that carbapenem monotherapy may no longer be an appropriate empirical choice for many patients with severe Gram-negative infections.^5^^,^^6^ Of particular concern is the increasing prevalence of carbapenem-resistant Enterobacterales (CRE) infections, for which treatment options are limited (see the related article by Bassetti and Garau in this Supplement). This has led to the development of new agents that combine a β-lactam antibiotic with a potent β-lactamase inhibitor (BLI), including three carbapenem/BLI combinations: meropenem/vaborbactam [Vabomere^®^ in the US (Melinta); Vaborem^®^ in Europe (Menarini)], imipenem/cilastatin/relebactam (Recarbrio^®^; Merck Sharp & Dohme) and meropenem/nacubactam (in early clinical development; Meiji Seika Pharma and Roche).

This article reviews the rationale for this type of combination in the treatment of patients with MDR Gram-negative infections, and describes the combinations currently available or in late-stage development, with a focus on the preclinical and clinical data for meropenem/vaborbactam.

## Rationale for carbapenem/β-lactam combinations

Carbapenems act by inducing the lysis of bacterial cells. These agents do not easily cross the outer membrane of Gram-negative organisms, but rely on transport by porins.^7^ Once inside the periplasmic space, the carbapenems acetylate the penicillin-binding proteins (PBPs) responsible for peptidoglycan formation in the cell wall. Inhibition of PBPs weakens the wall, leading to cell lysis and death.^7^

Many antibiotics have the same mechanism of action. but are broken down by β-lactamase enzymes in the periplasmic space before they can cause appreciable cell lysis. β-Lactamases fall into several categories based on their structure and hydrolytic activity (Table 1).^8^ Class A, C and D β-lactamases of the Ambler classification utilize active-site serine residues, whereas class B β-lactamases utilize zinc ions and are called metallo-β-lactamases (MBLs). The ability of the new β-lactamase inhibitor compounds to act on different enzymes is variable and is minimal against type B β-lactamases (MBLs). However, they are active against several clinically relevant class A and D enzymes [OXA-23, OXA-48, *Klebsiella pneumoniae* carbapenemase (KPC)].^7^ Currently, KPC-2 is the most widespread β-lactamase responsible for carbapenem resistance.^9^

**Table 1.** Nomenclature of clinically important enzymes^8^

Molecular (Ambler) class	Functional group or subgroup	Common name^a^	Clinically relevant enzyme(s) or enzyme family(ies)	Characteristic substrate profile^b^	Characteristic inhibitor profile^c^
A	2a	Penicillinase	PC1/*blaZ*	Narrow-spectrum PENs	CLA, TZB
A	2b	Penicillinase	TEM-1, SHV-1	Narrow-spectrum PENs, early CEPHs	CLA, TZB
A	2be	ESBL	TEM-10, SHV-2, CTX-M-15	Narrow-spectrum PENs, early CEPHs, ES-CEPHs, monobactams	CLA, TZB, AVI
A	2br	IRT	TEM-30 (IRT-2)	PENs, early CEPHs	TZB, AVI
A	2e	ESBL cephalosporinase	CepA	ES-CEPHs	CLA but not ATM
A	2f	Carbapenemase	KPC	All FDA-approved β-lactams	AVI, REL, VAB
A	2f	Carbapenemase	SME	PENs, early CEPHs, carbapenems, monobactams; not ES-CEPHS	CLA, AVI, VAB
B1, B3	3a	MBL, carbapenemase	IMP, NDM, VIM, SPM	All PENs, CEPHs, carbapenems; not monobactams	EDTA; no clinically approved inhibitor
B2	3b	MBL, carbapenemase	L1, CphA	Carbapenems preferred	EDTA; no clinically approved inhibitor
C	1	Cephalosporinase	AmpC	CEPHs	ATM, AVI, VAB
D	2d	Oxacillinase	OXA-1	PENs, especially oxacillin/cloxacillin	Variable
D	2df	Carbapenemase	OXA-23, OXA-48, OXA-181, OXA-232	PENs, especially oxacillin/ cloxacillin, carbapenems	AVI (OXA-48)

a ESBL, extended-spectrum β-lactamase; IRT, inhibitor-resistant TEM; MBL, metallo-β-lactamase.

b CEPH, cephalosporin; ES-CEPHS, expanded-spectrum cephalosporins; PEN, penicillin.

c ATM, aztreonam; AVI, avibactam; CLA, clavulanic acid; REL, relebactam; TZB, tazobactam; VAB, vaborbactam.

The recently developed combination antibiotics include both a carbapenem and a BLI with inhibitory activity against a range of β-lactamase enzymes, including several carbapenemases.^10–12^ However, these new agents have limited or no activity against MBLs, meaning that they will be less effective in regions where MBLs are prevalent.

## Carbapenem/β-lactamase inhibitor combinations

Currently, there are two carbapenem/BLI combinations commercially available: meropenem/vaborbactam and imipenem/cilastatin/relebactam. Meropenem/vaborbactam was approved by the US FDA in 2017 for the treatment of complicated urinary tract infection (cUTI) in adults,^13^ and by the EMA in 2018 for the treatment of cUTI (including pyelonephritis), complicated intra-abdominal infection (cIAI) or hospital-acquired pneumonia [HAP; including ventilator-assisted pneumonia (VAP)] in adults, as well as bacteraemia that occurs in association (or suspected association) with any of these infections.^14^ In the EU, meropenem/vaborbactam is also indicated for the treatment of infections due to aerobic Gram-negative organisms in adults with limited treatment options.^14^ Imipenem/cilastatin/relebactam was approved by the FDA in adults with limited or no alternative treatment options for treatment of cUTI or cIAI in 2019,^15^ and for treatment of HAP and VAP in 2020,^16^ and by the EMA for the treatment of infections due to aerobic Gram-negative organisms in adults with limited treatment options in 2020.^17^ A third combination, meropenem/nacubactam, is being developed by NacuGen Therapeutics and is in early clinical development.^18^

The BLIs in these three combination agents do not have a β-lactam structure. Relebactam and nacubactam are diazabicyclooctane molecules, both structurally related to avibactam (approved for use in combination with ceftazidime),^12^^,^^19^ whereas vaborbactam is a cyclic boronic acid molecule.^10^^,^^12^ All three BLIs have a similar spectrum of activity against β-lactamase enzymes (Table 2), including KPC,^12^^,^^20^ and have shown *in vivo* activity against KPC and other class A carbapenemases.^21–24^

**Table 2.** Inhibitory activity of vaborbactam, relebactam and nacubactam against various β-lactamase enzymes^12^^,^^20^

β-Lactamase	Vaborbactam	Relebactam	Nacubactam
Class A enzymes			
TEM	+	+	+
SHV	+	+	NA
CTX-M	+	+	+
KPC	+	+	+
Class B enzymes			
MBL	–	–	
IMP	NA	NA	–
Class C enzymes			
AmpC	+	+	+
CMY-2	NA	NA	+
Class D enzymes			
OXA	–^a^	+/–	+/–

–, No inhibitory activity; +, inhibitory activity; NA, not available.

a Limited data available.

The carbapenems in the combinations are similarly effective in the management of serious infections, but meropenem shows greater activity than imipenem against Gram-negative bacilli, whereas imipenem is generally more active than meropenem against Gram-positive cocci.^25^ Imipenem is always administered with cilastatin, because it is rapidly inactivated by renal dehydropeptidase I when administered alone. Cilastatin is a renal dehydropeptidase I inhibitor, which prolongs the half-life of imipenem and reduces the risk of renal toxicity.^26^

In 2019, meropenem/vaborbactam was added to WHO’s list of essential medicines^27^ because of its activity against priority 1 antibiotic-resistant pathogens. The rest of this article focuses on the research with this agent.

## Meropenem/vaborbactam

### Preclinical studies

*In vitro* data show that meropenem/vaborbactam is highly active against a range of KPC-positive CRE at concentrations of ≤4 mg/L (the susceptibility breakpoint defined by the US FDA) or ≤8 mg/L (the breakpoint defined by EUCAST).^28–30^ These include *K. pneumoniae, Escherichia coli, Enterobacter* spp. (including *Enterobacter cloacae*), *Klebsiella oxytoca, Serratia marcescens and Citrobacter* spp.^28–30^ The overall MIC required to inhibit 90% of isolates (MIC_90_) for meropenem/vaborbactam was ≤1 mg/L for all isolates,^28^^,^^29^ including isolates harbouring the KPC-2 and KPC-3, AmpC, CTX-M and SHV enzyme variants.^28^^,^^30^ In a comparative analysis, meropenem/vaborbactam showed more potent *in vitro* antimicrobial activity than meropenem alone, ceftazidime/avibactam, tigecycline, ceftazidime alone, minocycline, gentamicin or polymyxin B against clinical isolates of KPC-positive Enterobacterales from a global collection.^28^ Across all the isolates tested, the MIC_90_ of meropenem/vaborbactam was 1 mg/L, which was four times more potent than ceftazidime/avibactam and >64 times more potent than meropenem alone.^28^

**Figure 1.**
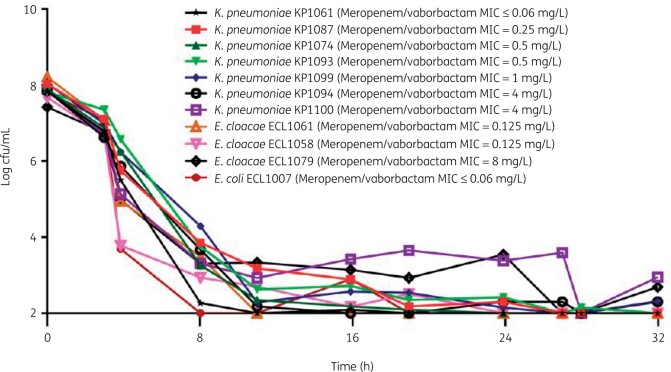
Activity of meropenem/vaborbactam against KPC-containing Enterobacterales at concentrations simulating those expected after administration of the approved human dosage (2 g meropenem/2 g vaborbactam administered by 3 h IV infusion every 8 h).^31^ MICs refer to the meropenem/vaborbactam MIC with vaborbactam 8 mg/L. cfu, colony-forming units; IV, intravenous; MIC, minimum inhibitory concentration. Reproduced with permission from Sabet *et al*.^31^ (Figure 3).

In an *in vitro* hollow-fibre model, meropenem/vaborbactam at concentrations equivalent to those achieved by administration of the approved human dosage showed significant activity against a range of KPC-producing CRE strains, including *K. pneumoniae, E. cloacae and E. coli* isolates (Figure 1).^31^ Importantly, the isolates used in this study included strains with a range of KPC enzymes and with porin mutations that could confer meropenem resistance. Meropenem/vaborbactam was active against all strains except KP1092 and KP1254, which harboured loss-of-function mutations in the genes for OmpK36 porins.^31^ Data from that study and another^32^ indicate that strains with non-functional or poorly functional OmpK35 or OmpK36 porins are the least susceptible to meropenem/vaborbactam, as are those with an increase in *bla*_KPC_ gene copy number.^31^^,^^32^ These are the same mechanisms that confer resistance to ceftazidime/avibactam,^32^ but meropenem/vaborbactam has shown potent activity against isolates resistant to ceftazidime/avibactam.^33^^,^^34^ In fact, the *in vitro* data suggest that, at concentrations simulating exposure after human dosing, meropenem/vaborbactam retains activity against CRE strains that harbour these resistance mechanisms,^31^ so there is a low risk of resistance development with clinical use of meropenem/vaborbactam.^33–35^ The increasing rates of reported ceftazidime/avibactam resistance may make meropenem/vaborbactam the preferred agent for KPC infections, especially considering the possible lower risk of selection for cross-resistance.^36^ However, meropenem/vaborbactam resistance was found in 8% (5/62) of the KPC-producing strains isolated from patients with bloodstream infections in an Italian series.^37^ Molecular characterization revealed that resistance was due to porin mutations and was associated with reduced susceptibility to both ceftazidime/avibactam and carbapenems.^37^

The *in vivo* activity of meropenem/vaborbactam was demonstrated in a range of murine models of infection, including thigh infections, lung infections or pyelonephritis caused by KPC-producing CRE,^32^^,^^38^ and a neutropenic thigh infection caused by *P. aeruginosa and A. baumannii*.^39^ In all of these models, meropenem/vaborbactam demonstrated significant bacterial killing,^31^^,^^38^^,^^39^ providing support for clinical investigation.

### Pharmacokinetic studies

Studies in healthy volunteers demonstrated that meropenem and vaborbactam have similar pharmacokinetic properties.^13^^,^^14^^,^^22^^,^^40–43^ Both compounds are widely distributed throughout the body and are rapidly eliminated, predominantly through renal excretion.^41^^,^^42^

The clinically tested doses of meropenem and vaborbactam were chosen based on pharmacokinetic and pharmacodynamic studies in animal and *in vitro* models of infection. The studies in *in vitro* models of infection allowed for detection of resistance, which is not usually possible in animal models of infection, and thus identified conditions and pharmacodynamic parameters associated with resistance prevention.^35^ The resulting dosage regimen that accomplished this objective was meropenem 2 g plus vaborbactam 2 g administered as a 3 h infusion every 8 h. This dose of meropenem/vaborbactam maximizes the percentage of the dosing interval during which free-drug levels exceed the MIC for the target organisms, based on the time-dependent killing profile of meropenem.^22^ This may minimize the likelihood of resistance developing in strains that are not susceptible to meropenem alone.

The closely matched pharmacokinetic profiles of meropenem and vaborbactam in humans (including the shared route of excretion) result in comparable changes in pharmacokinetics in patients with renal impairment and similar recommended changes in dose in these patients.^41^ Dose adjustment is required in patients with an estimated glomerular filtration rate of <50 mL/min/1.73 m^2^ or creatinine clearance (CL_CR_) ≤39 mL/min.^13^^,^^14^ The high rate of clearance with continuous renal replacement therapy necessitates prolonged infusion dosing, in addition to dosage adjustments. The usual dose of meropenem/vaborbactam is 2 g/2 g every 8 h, but patients with a CL_CR_ of 20–39 mL/min should receive 1 g/1 g every 8 h, those with a CL_CR_ of 10–19 mL/min should receive 1 g/1 g every 12 h, and those with a CL_CR_ of <10 mL/min should receive 0.5 g/0.5 g every 12 h.^14^ According to simulations, these doses will achieve the target pharmacokinetic/pharmacodynamic drug exposures in >90% of patients.^44^

The combination shows good penetration of lower respiratory tract tissues after intravenous (IV) administration, with epithelial lining fluid concentrations of 65% for meropenem and 79% for vaborbactam (based on free drug).^43^ Given the efficacy of meropenem/vaborbactam in patients with serious CRE infections in the TANGO II trial, including those with HAP/VAP (described in the next section),^45^ the ability of meropenem/vaborbactam to enter the bronchial epithelial lining fluid is likely to translate into a clinical benefit for patients with HAP/VAP, and to reduce the risk of resistance development in this population.

Based on the well-established pharmacokinetic profile of meropenem and the pharmacokinetic profile of vaborbactam defined during Phase I development, the clinical development of meropenem/vaborbactam was able to proceed without the need for Phase II (dose-finding) studies.

### Clinical studies

Because Phase II studies were not required for meropenem/vaborbactam, the efficacy and safety of the combination was able to be investigated in two sequential Phase III studies: TANGO I in patients with cUTI and acute pyelonephritis (AP)^46^ and TANGO II in patients with serious CRE infections.^45^

TANGO I was the first study to investigate the efficacy of the proposed meropenem/vaborbactam dosage in a population of patients with complicated infections. This multicentre, randomized, double-blind, non-inferiority study compared meropenem/vaborbactam with piperacillin/tazobactam in 550 patients with cUTI or AP, with Enterobacterales as the most common causative pathogens.^46^

Patients in both groups were treated with IV agents for 1–15 days (mean 8 days), but the overall mean antibiotic treatment duration was 10 days when oral step-down therapy was included. Two different primary endpoints were defined for the two key regulatory bodies. For the US FDA, the primary endpoint was overall success at the end of IV treatment, defined as a composite of clinical cure (complete resolution or significant improvement in symptoms) and microbial eradication (<10^4^ cfu in urine). For the EMA, the primary endpoint was microbial eradication (<10^3^ cfu in urine) at the test-of-cure visit (7 days after the end of treatment).^46^

The non-inferiority of meropenem/vaborbactam was demonstrated for both primary endpoints. The rate of overall success (US FDA criterion) was 98.4% in the group receiving meropenem/vaborbactam and 94.0% in the group receiving piperacillin/tazobactam (difference 4.5%). Because the lower limit of the 95% CI was greater than the prespecified non-inferiority margin of −15%, meropenem/vaborbactam was shown to be significantly non-inferior to piperacillin/tazobactam (*P < *0.001). Additionally, because the lower limit of the 95% CI was also greater than 0%, meropenem/vaborbactam was also shown to be superior to piperacillin/tazobactam (*P = *0.01).^46^

Microbial eradication (EMA criterion) was seen in 66.7% of patients treated with meropenem/vaborbactam and 57.7% of patients treated with piperacillin/tazobactam (difference 9.0%, *P < *0.001 for non-inferiority). Similar rates of overall success were seen in subgroups of patients with AP and cUTI with or without a non-removable source of infection. Patients with bacteraemia all showed negative cultures after treatment.^46^ Secondary endpoints (overall success at test of cure, clinical cure at the end of IV treatment and microbial eradication at test of cure) all showed a similar pattern, with comparable or slightly higher rates of each outcome in the meropenem/vaborbactam group compared with piperacillin/tazobactam. The incidence and type of adverse events were similar in the two groups.^46^ The incidence and type of adverse events with meropenem/vaborbactam in TANGO I were similar to those seen previously with meropenem, which suggests that adding vaborbactam does not significantly alter the safety profile of meropenem.

TANGO II was the first randomized and controlled clinical trial that tested the efficacy and safety of an antibiotic in a population with infections caused by CRE and CRE-KPC pathogens, including immunocompromised patients with several comorbidities, who are usually excluded from clinical trials.^45^^,^^47^ This pathogen-specific, randomized, controlled trial compared meropenem/vaborbactam as a single agent with best-available therapy (BAT), usually administered as a combination of multiple antibiotics, in 77 patients with CRE infections including bacteraemia (36%), cUTI or AP (45.3%), cIAI (9.3%) and HAP or VAP (9.3%).^45^ The most common pathogen in the intent-to-treat population was KPC-producing *K. pneumoniae* with a high-level of meropenem resistance (MIC_50_ of 64 mg/L in both groups).

Patients were randomized 2:1 to open-label treatment with meropenem/vaborbactam or BAT selected by the investigator; this could include any monotherapy or combination of polymyxins, carbapenems, aminoglycosides or tigecycline, or monotherapy with ceftazidime/avibactam.^45^ An interim analysis showed that the risk/benefit profile favoured meropenem/vaborbactam, so the independent Data Safety Monitoring Board recommended stopping randomization to the BAT group, leading to early discontinuation of study recruitment.

**Figure 2.**
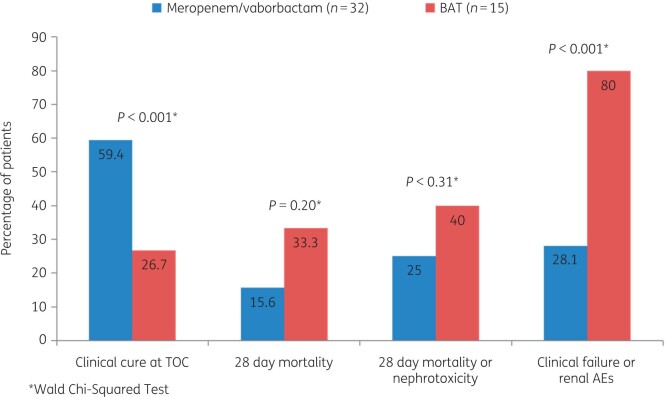
Outcomes in patients with carbapenem-resistant Enterobacterales infections who received meropenem/vaborbactam or best-available therapy (BAT) in the TANGO II study.^45^ AEs, adverse events; TOC, test of cure.

Among patients with a confirmed CRE infection, clinical cure rates were significantly higher in the group receiving meropenem/vaborbactam compared with BAT [65.6% versus 33.3% at the end of treatment (*P = *0.03) and 59.4% versus 26.7% at test of cure (*P = *0.02)]. The 28 day mortality rate was numerically lower with meropenem/vaborbactam (15.6%) than with BAT (33.3%; *P = *0.20) (Figure 2); only one of the five deaths in the meropenem/vaborbactam group was related to sepsis compared with four of the five deaths in the BAT group. The difference in all-cause mortality at Day 28 across all indications was driven by mortality differences in subjects with HAP/VAP or bacteraemia, the sickest subjects enrolled in the study.^45^

Moreover, meropenem/vaborbactam was associated with fewer adverse events (Figure 2), including severe, serious, and drug-related adverse events, compared with BAT. The difference in renal events was notable, with 24.0% of patients in the BAT group developing renal-related adverse events compared with 4.0% in the meropenem/vaborbactam group; the incidence of acute renal failure was 12.0% with BAT versus 2.0% with meropenem/vaborbactam.^45^

Although TANGO II was a randomized study, patients with prior antibiotic failure, who are expected to have a lower response to salvage therapy, were all enrolled in the meropenem/vaborbactam group. Therefore, a *post hoc* analysis was conducted in patients who had not received prior antibiotics (i.e. the population receiving meropenem/vaborbactam or BAT as first-line therapy for CRE).^48^ This analysis showed more marked differences in clinical cure and microbiological cure rates between the meropenem/vaborbactam and the BAT group than was seen in the overall population. These data confirm that, in critically ill patients, prompt initiation of the antibiotic therapy is linked to better outcomes. First-line use of meropenem/vaborbactam was associated with a clinical cure rate of 82.6% at end of treatment and 69.6% at test of cure compared with 33.3% and 26.7% in the first-line BAT group, representing an improvement of 49.3% and 42.9% at these timepoints, respectively.^48^ Because the incidence of renal adverse events was higher in the BAT group, the risk/benefit profile clearly favoured meropenem/vaborbactam.

### Real-world clinical use

Importantly, three real-world observational studies conducted in the USA have confirmed the effectiveness and safety of meropenem/vaborbactam during real-world clinical use.^49–51^ The first was a single-centre prospective observational study conducted in 20 consecutive patients who received meropenem/vaborbactam between December 2017 and April 2019.^49^ The other two were multicentre, retrospective studies: one in 40 patients with Gram-negative infections between October 2017 and June 2019,^50^ and the other in 131 patients with CRE infections treated with either meropenem/vaborbactam (*n = *26) or ceftazidime/avibactam (*n = *105) between February 2015 and October 2018.^51^

In the single-centre study, patients had bacteraemia (*n = *8), pneumonia (*n = *6; VAP in 5/6), tracheobronchitis (*n = *2; ventilator-associated in 1/2), skin and soft tissue infections (*n = *2), pyelonephritis (*n = *1) and peritonitis with intra-abdominal abscess (*n = *1). This was a vulnerable group of patients: 14 (70%) were in the ICU at the onset of the infection, and seven (35%) required renal replacement therapy with intermittent or continuous haemodialysis. In addition, the median Acute Physiology and Chronic Health Evaluation (APACHE II) score was 20 and median Charlson comorbidity index (CCI) was 4. The most common pathogen was *K. pneumoniae*, and 19 of the 20 isolates (95%) were resistant to ertapenem.^49^

Patients in the multicentre studies were similarly vulnerable. In the study of patients with Gram-negative infections, median APACHE II score was 17, median CCI was 6, 70% were in the ICU, and 90% had at least one risk factor for developing an MDR infection.^50^ In that study, the most common infection was pneumonia (*n = *13), followed by UTI (*n = *8), intra-abdominal infections (*n = *5) and skin and soft tissue infections (*n = *5); 11 patients had bacteraemia—primary in 2 and secondary in 9. Overall, 45 pathogens were isolated, most commonly *K. pneumoniae* (47%), *E. cloacae* (20%) and *E. coli* (13%).^50^ In the study of patients with CRE infections, 57% of patients were in ICU, 41% had bacteraemia and median APACHE II scores were 26 and 27 in the meropenem/vaborbactam and ceftazidime/avibactam groups, respectively.^51^ In that study, the most commonly identified CRE organisms were *Klebsiella* spp. (69%) and other Enterobacterales (21%).^51^

All three studies defined clinical success as the composite of 30 day survival, absence of recurrence at 30 days after starting treatment, and resolution of signs and symptoms of infection while receiving treatment.^49–51^ This endpoint was achieved by 13/20 patients (65%) in the single-centre study,^49^ by 28/40 patients (70%) in the study of patients with Gram-negative infections,^50^ and by 18/26 patients (69%) with meropenem/vaborbactam and 65/105 patients (62%) with ceftazidime/avibactam in the study of patients with CRE infections.^51^ In patients with Gram-negative infections, better clinical success was seen in patients with a community-acquired infection (86%) than a nosocomial infection (50%).^50^ Severe adverse events were reported in one patient in the single centre study (eosinophilia)^49^ and one in the study of patients with Gram-negative infections (Stevens-Johnson syndrome).^50^ In the study of patients with CRE infections, the incidence of nephrotoxicity was 14% with meropenem/vaborbactam and 29% with ceftazidime/avibactam.^51^

The management of Gram-negative infections is often complicated by the presence of multiple morbidities and need of venous access devices, catheters or ventilators in fragile subjects. Real-world case reports indicate that meropenem/vaborbactam can be effective even in these vulnerable individuals.^47^^,^^52^ Jorgensen *et al*.^47^ described the successful use of meropenem/vaborbactam to treat bacteraemia caused by carbapenem-resistant *S. marcescens* and carbapenem-resistant *Enterobacter aerogenes* in an asplenic patient with HIV infection and renal failure requiring dialysis. In another case, Athans and colleagues^52^ used meropenem/vaborbactam to treat a subphrenic abscess and persistent bacteraemia caused by KPC-producing carbapenem-resistant *K. pneumoniae* in a liver transplant recipient who developed hepatic artery thrombosis and graft failure. The infection had not responded to courses of ceftazidime/avibactam or polymyxin B, initially administered as monotherapy, then with added gentamicin and tigecycline. This combination was discontinued because of renal toxicity. Once meropenem/vaborbactam was started, the patient’s renal function improved and their infection cleared, allowing the patient to undergo a successful second transplantation.^52^ The authors of both these reports highlighted the advantage of being able to use a single agent rather than combination therapy to treat CRE infections in clinically complex patients, thereby reducing the risk of toxicity.^47^^,^^52^

## Conclusions

Given the increasing threat posed by carbapenem-resistant pathogens and the limited treatment options for patients with CRE infections, the development of these new combinations of carbapenems and BLIs represents an important therapeutic advance. Meropenem/vaborbactam demonstrates considerable *in vitro and in vivo* activity against these pathogens, with a low potential for resistance at clinically relevant doses. The available clinical data show its efficacy and tolerability in patients with complicated CRE infections, with a low potential for toxicity compared with the combination regimens that have been the standard of care until now. In addition, observational studies and case reports have confirmed the efficacy of meropenem/vaborbactam in a real-world setting, including in very clinically complex patients with immunosuppression, renal dysfunction, or extensively drug-resistant organisms. Therefore, it is not surprising that this agent is now listed by the WHO as an essential medicine. With time, further data will emerge about how to optimally use meropenem/vaborbactam and other carbapenem-BLI combinations in the treatment of CRE.

## Transparency declarations

E.B. has participated in advisory boards and received payment for conferences from Menarini.

Catherine Rees of Springer Healthcare Communications wrote the first draft of this manuscript. This medical writing assistance was funded by A. Menarini Farmaceutica Internazionale. This paper was published as part of a Supplement sponsored and financially supported by A. Menarini Farmaceutica Internazionale.
